# Nanosynthesis of Silver-Calcium Glycerophosphate: Promising Association against Oral Pathogens

**DOI:** 10.3390/antibiotics7030052

**Published:** 2018-06-27

**Authors:** Gabriela Lopes Fernandes, Alberto Carlos Botazzo Delbem, Jackeline Gallo do Amaral, Luiz Fernando Gorup, Renan Aparecido Fernandes, Francisco Nunes de Souza Neto, José Antonio Santos Souza, Douglas Roberto Monteiro, Alessandra Marçal Agostinho Hunt, Emerson Rodrigues Camargo, Debora Barros Barbosa

**Affiliations:** 1 Department of Dental Materials and Prosthodontics, School of Dentistry, Araçatuba, São Paulo State University (UNESP), Araçatuba 16015-050, São Paulo, Brazil; fernandesgabriela@hotmail.com (G.L.F.); renanfernandes@fai.com.br (R.A.F.); 2 Department of Pediatric Dentistry and Public Health, School of Dentistry, Araçatuba, São Paulo State University (UNESP), Araçatuba 16015-050, São Paulo, Brazil; adelbem@foa.unesp.br (A.C.B.D.); jackelineamaral@gmail.com (J.G.d.A.); joseanonio_249@hotmail.com (J.A.S.S.); 3 Department of Chemistry, Federal University of São Carlos, São Carlos 13565-905, São Paulo, Brazil; lfgorup@gmail.com (L.F.G.); francisco_nsn@yahoo.com.br (F.N.d.S.N.); camargo@ufscar.br (E.R.C.); 4 FACET—Department of Chemistry, Federal University of Grande Dourados, Dourados 79804-970, Mato Grosso do Sul, Brazil; 5 Department of Dentistry, University Center of Adamantina (UNIFAI), Adamantina 17800-000, São Paulo, Brazil; 6 Graduate Program in Dentistry (GPD—Master´s Degree), University of Western São Paulo (UNOESTE), Presidente Prudente 19050-920, São Paulo, Brazil; douglasmonteiro@hotmail.com; 7 Department of Microbiology and Molecular Genetics, Michigan State University, East Lansing, MI 48823, USA; alehunt@msu.edu

**Keywords:** silver, calcium glycerophosphate, nanoparticles, *Candida albicans*, *Streptococcus mutans*

## Abstract

Nanobiomaterials combining remineralization and antimicrobial abilities would bring important benefits to control dental caries. This study aimed to produce nanocompounds containing calcium glycerophosphate (CaGP) and silver nanoparticles (AgNP) by varying the reducing agent of silver nitrate (sodium borohydride (B) or sodium citrate (C)), the concentration of silver (1% or 10%), and the CaGP forms (nano or commercial), and analyze its characterization and antimicrobial activity against ATCC *Candida albicans* (10231) and *Streptococcus mutans* (25175) by the microdilution method. Controls of AgNP were produced and silver ions (Ag^+^) were quantified in all of the samples. X-ray diffraction, UV-Vis, and scanning electron microscopy (SEM) analysis demonstrated AgNP associated with CaGP. Ag^+^ ions were considerably higher in AgCaGP/C. *C. albicans* was susceptible to nanocompounds produced with both reducing agents, regardless of Ag concentration and CaGP form, being Ag10%CaGP-N/C the most effective compound (19.5–39.0 µg Ag mL^−1^). While for *S. mutans*, the effectiveness was observed only for AgCaGP reduced by citrate, also presenting Ag10%CaGP-N the highest effectiveness (156.2–312.5 µg Ag mL^−1^). Notably, CaGP enhanced the silver antimicrobial potential in about two- and eight-fold against *C. albicans* and *S. mutans* when compared with the AgNP controls (from 7.8 to 3.9 and from 250 to 31.2 µg Ag mL^−1^, respectively). The synthesis that was used in this study promoted the formation of AgNP associated with CaGP, and although the use of sodium borohydride (B) resulted in a pronounced reduction of Ag^+^, the composite AgCaGP/B was less effective against the microorganisms that were tested.

## 1. Introduction

The synthesis and study of properties of new biomaterials has been emphasized lately with the improvement of nanotechnology. In this context, the development of nanomaterials has been the focus of many areas of chemistry, physics, and materials science because of the promising characteristics that these materials exhibit [1].

Nanotechnology aims to manipulate particles by creating new structures with favorable properties in many areas, such as medicine and dentistry [2], and new alternatives of treatment for oral pathologies are emerging. Metallic nanoparticles, in particular silver nanoparticles (AgNP), have been studied as an alternative antimicrobial agent against a broad spectrum of species in the control of oral biofilms [3,4,5]. Although there are several studies where AgNP are used as antimicrobial agents, their mechanism of action is not completely understood. Kim et al. [6] and Besinis et al. [4] related their antimicrobial action to the toxicity resulting from free metal ions dissolution from the surface of the AgNP. In addition, AgNP would lead to oxidative stress through the generation of reactive oxygen species (ROS), interacting with cytoplasmic and nucleic acid components by inhibiting enzymes of the respiratory chain and changing the permeability of the cytoplasmatic bacterial membrane [7,8,9,10,11]. 

Among oral pathologies, dental caries is one of the most common diseases in humans that relates to genetics, saliva, and diet of the host [9]. *Streptococcus mutans* is the main cariogenic microorganism owing to its ability to produce acids and glucans from sugar metabolism, which exceed the buffering capacity of saliva [9,10,11] and leads by a localized and irreversible destruction of the tooth structure [9,12]. However, recent evidence indicates the presence of *C. albicans* and *S. mutans* in oral biofilms, suggesting that the interaction between them can lead to the development of caries [9,13,14]. *C. albicans* colonization depends on the presence of the bacteria, which, besides promoting adhesion sites, act as a carbon source for yeast growth. On the other hand, yeasts reduce the levels of oxygen for streptococci [9]. Studies have shown the resistance of many microorganisms to antimicrobial agents currently used [15,16].

Studies since the 1930s [17] have reported the importance of using calcium phosphate derivatives for favouring the remineralization process in dental caries. Calcium glycerophosphate (CaGP) is an organic phosphate salt with anti-caries properties being demonstrated in studies carried out in monkeys [18] and in rats [19]. It is action in dental biofilms may be related to the increase of calcium and phosphate levels [20], buffering capacity [18], and reduction of the mass of the biofilms [21]. Becauses that it seems to interact with dental tissues [22], CaGP has been incorporated in dentifrices [23,24]. Do Amaral et al. [25] and Zaze et al. [26], when associating CaGP (0.25%) in toothpastes with fluoride at low concentrations, found the same efficacy against caries in enamel when compared to dentifrices that were supplemented with a higher concentration of fluoride demonstrating CaGP be an good option for oral products to both prevent caries and avoid fluorose in dental tissues.

The use of a biomaterial containing both an antimicrobial and a compound acting as a source of calcium phosphate for dental remineralization would have a great impact on the prevention and control of dental caries. Therefore, this study aimed to produce nanocompounds containing calcium glycerophosphate (CaGP) and silver nanoparticles (AgNP) by varying the reducing agent of silver nitrate (sodium borohydride or sodium citrate), the concentration of silver (1% or 10%), and the CaGP forms (nano or microparticulated), and analyze its characterization and antimicrobial activity against ATCC strains of *Candida albicans* and *Streptococcus mutans*.

## 2. Results

### 2.1. Synthesis and Characterization of Ag-CaGP Nanocomposites

**Figure 1:**
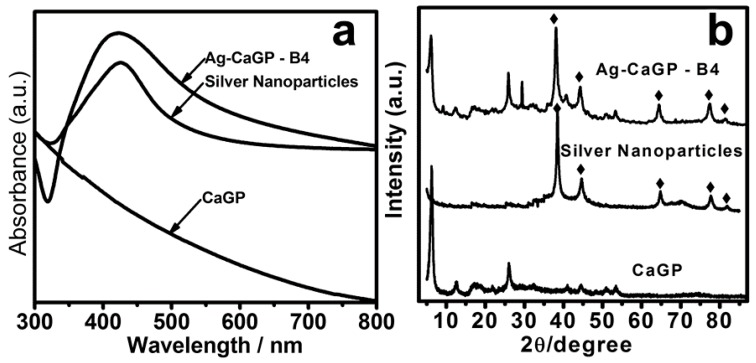
(**a**) UV-Vis (**b**) XRD pattern of Ag-CaGP (B4 group) nanocomposite, silver nanoparticles, and CaGP.

UV-Vis absorption spectroscopy (UV-Vis) showed that Ag-CaGP nanocomposites presented silver in nanosized dimensions in all of the nanocomposites synthesized, regardless of the reducing agent used. It was demonstrated by the presence of an intense absorption peak, denominated plasmonic band, which occurred between 420 and 450 nm (Figure 1a, Figure S1). It characterizes noble metal nanoparticles, with strong absorption band being observed in the visible region [27]. The CaGP did not exhibit absorption peak in the visible region of the electromagnetic spectrum.

X-ray diffraction (XRD) pattern indicated that all of the Ag-CaGP nanocomposites were composed of AgNP and CaGP for confirming the presence of silver in Ag-CaGP nanocomposites through comparison of the nanoparticles and CaGP. The typical powder XRD pattern of the prepared CaGP showed diffraction peaks at 2θ = 6.30°, 12.3°, 26.4°, 41.1°, and 44.2° (Figure 1b, Figure S2), and the corresponding crystallographic form (PDF No. 1-17) [28]. The typical powder XRD pattern of the silver nanoparticles showed (Figure 1b) diffraction peaks at 2θ = 38.2°, 44.4°, 64.6°, 77.5°, and 81.7°, which can be indexed to (111), (200), (220), (311), and (222) planes of pure silver with face-centered cubic system (PDF No. 04-0783).

**Figure 2:**
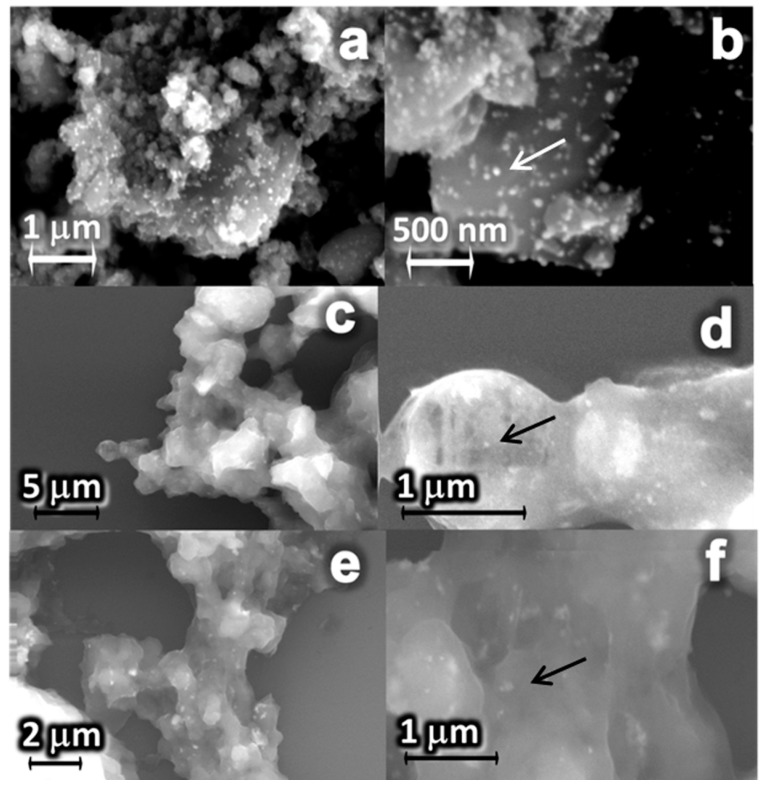
SEM images of the Ag-CaGP nanocomposites: B4 (**a**,**b**), C4 (**c**,**d**), and B8 (**e**,**f**) at different magnifications. The arrows indicate silver nanoparticles on the surface of CaGP in B4 and in the bulk of CaGP in C4 and B8.

Nanostructured materials that exhibit a pattern of small nanoparticles scattered on a larger surface, similar to glass bead embellishments on a Christmas tree, are generally classified as a decorated material. The scanning electron microscopy (SEM) images of Figure 2 show this typical pattern, with spherical silver nanoparticles (indicated by arrows) decorating the surface of the CaGP microparticles in all synthesized nanocomposites containing 10% Ag (B4; B8; C4). In addition, transmission electron microscopy (TEM) was performed for the nanocomposite B4 (Figure S3).

**Figure 3:**
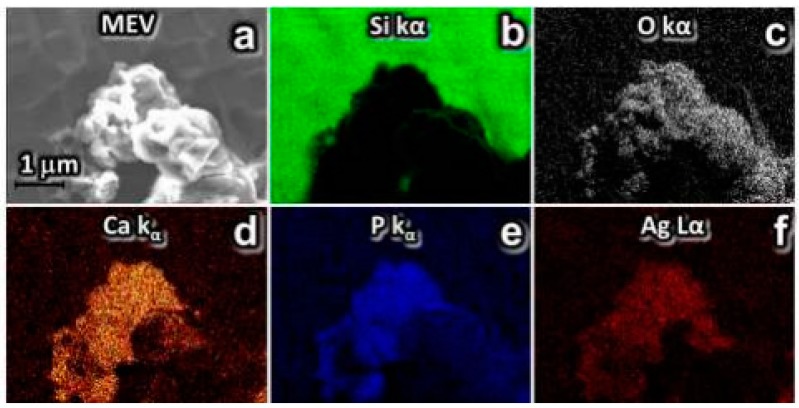
SEM and EDS mapped in 2D elements issuance Si Kα, O Kα, P Kα, Ca Kα, and Ag Kα false color. Analysis of the distribution of silver nanoparticles on the Ag-CaGP for sample B4: (**a**) SEM image; (**b**) chemical mapping of silicon element present in the substrate, where the electron beam was focused directly on the substrate and is showed in green color, and the dark regions the beam was focused in Ag-CaGP nanocomposite B4; (**c**–**f**) oxygen, calcium, phosphorus, and silver, respectively, demonstrating they are constituents of the Ag-CaGP.

**Figure 4:**
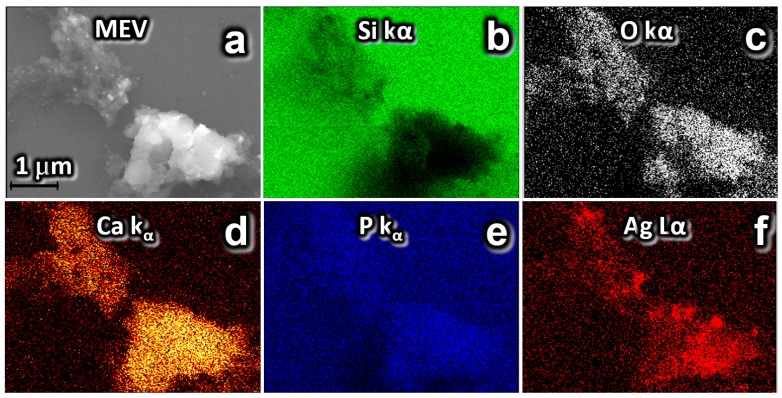
SEM and EDS mapped in 2D elements issuance Si Kα, O Kα, P Kα, Ca Kα, and Ag Kα false color. Analysis of the distribution of silver nanoparticles on the Ag-CaGP for sample C4: (**a**) SEM image; (**b**) chemical mapping of silicon element present in the substrate, where the electron beam was focused directly on the substrate and is showed in green color, and the dark regions the beam was focused in Ag-CaGP nanocomposite C4; and, (**c**–**f**) oxygen, calcium, phosphorus, and silver, respectively, demonstrating they are constituents of the Ag-CaGP.

The energy-dispersive X-ray sprectroscopy (EDS) clearly showed the outline of Ag-CaGP nanocomposites in all micrographs. Also, Figure 3 (B4), Figure 4 (C4) and Figures S4–S6, the two-dimensional (2D) images were constructed by analyzing the energy released from the issuance Si Kα, O Kα, P Kα, Ca Kα, and Ag Kα, indicating the distribution of these elements on the demarcated area in the micrograph. 

### 2.2. Minimum Inhibitory Concentration

The results showed that the MIC values were related to the synthesis process and the Ag concentration used (Table 1). Nanocomposites that were obtained using Na_3_C_6_H_5_O_7_ as reducing agent showed the most effective antimicrobial activity against *C. albicans* and *S. mutans*. In these composites, the lowest MIC values were observed for those containing 10% of Ag (C3 and C4), being between 19.05 and 39.05 µg/mL for *C. albicans* and 156.2 and 625 µg/mL for *S. mutans*. The nanocomposites that were synthesized using NaBH_4_ as reducing agent and isopropanol as solvent showed fungicidal effect varying between 100 and 1600 µg/mL, whilst no effect against *S. mutans* was observed. While the nanocomposites synthesized using the same reducing agent and deionized water as solvent did not show any effect against both microorganisms. In addition to the MICs found for the synthesized compounds, it was carried out the microdilution assay to find the MIC values for the solutions containing only AgNP or CaGP diluted in deionized water, besides the other compounds used in the synthesis reaction as reducing and surfactant agents. These data are showed in Table 2.

### 2.3. Determination of Ag^+^ Concentration

The Ag^+^ concentration of all the nanocomposites containing Ag (AgNP and Ag-CaGP) is showed in Table 1. For samples that were obtained through NaBH_4_ route (B1–B8), a reduction of ionic silver higher than 98% was observed, when considering the total amount of ionic silver added to the reaction was 500 µg Ag^+^/mL for B1, B2, B5, B6, and 5000 µg Ag^+^/mL for B3, B4, B7, and B8. While for the compounds that were synthesized using Na_3_C_6_H_5_O_7_ as reducing agent, the ionic silver remaining was higher and reached about 10% in those samples that were produced using initially 5000 µg Ag^+^/mL in the reaction process (C3 and C4). C1 and C2 presented 61.1% and 33.3%, respectively, of ionic silver in samples as the total Ag^+^ added in the reaction was 500 µg Ag^+^/mL. For AgNP with no CaGP added to the reaction (Table 2) obtained by Na_3_C_6_H_5_O_7_ route (nanoAg(Na_3_C_6_H_5_O_7_)) the Ag^+^ concentration was 107.25 µg/mL, whereas for AgNP produced through NaBH_4_ (nanoAg(NaBH_4_)) the Ag^+^ concentration was 576.19 µg/mL.

## 3. Discussion

In the present study, both of the synthesis methods proposed using sodium citrate or sodium borohydride as reducing agents, led to the anchorage between the silver nanoparticles and calcium glycerophosphate (Figure 2). Besides, in general, the nanocomposites (AgCaGP) were effective against reference strains of *Candida albicans* and *Streptococcus mutans*. Notably, CaGP substantively increased the antimicrobial effectiveness of silver in the AgCaGP, reducing up to a quarter their minimum inhibitory concentration when compared to the respective AgNP controls (Table 1 and Table 2).

Although the CaGP has been previously nanoparticulated before the Ag-CaGP synthesis, in our study it was not characterized as being in nanoparticulated form when associated with silver. It might be happen due to the poor solubility of calcium at pH = 7 [29], even when using the same dispersant (NH-PM), as preconized by Miranda et al., whom synthesized AgNP with hydroxyapatite. A pastier bulk was particularly noted in micrographics of Ag-CaGP when water was used as solvent instead of isopropanol (Figure 2c–f), regardless of the reducing agent that was used in the reaction. Although there has not been difference between micro and nanoparticulated-CaGP in the SEM images, its form influenced the amount of silver ions in the compounds (Table 1). In addition, our results showed the antimicrobial effectiveness against *C. albicans* and *S. mutans* for the samples of group C, and it could be explained by the highest amount of silver ions that are present in those compounds [4,30,31,32,33,34,35].

This expressive difference in the quantity of silver ions between groups B and C would be related to the characteristics of the reducing agents used, being sodium borohydride considered a stronger reducing agent than sodium citrate [33]. Although silver ions are effective to kill several pathogenic microorganisms, they are easily dispersed, which quickly decreases its local concentration to levels of low effectivity. Moreover, ambient light reduces ionic silver forming typical black spots on skin or on any surface of contact [36]. This process causes aesthetic problems and it has potential to injure healthy living tissues. Silver nanoparticles, contrary to ionic silver, induce the production of reactive oxygen species (ROS), which is the primary antimicrobial mechanism [37]. However, AgNP tend to form aggregates in the absence of any support, reducing their efficacy. Therefore, substrates decorated with immobilized AgNP exhibits enhanced antimicrobial activity for longer periods, reducing the undesirable secondary effects that are associated to free ionic silver [38]. Although the difficult to separate the impact of free ionic silver from the AgNP antimicrobial action, differences that were observed in minimum inhibitory concentrations (MIC), as shown in Table 1, for *C. albicans* and *S. mutans* suggests the influence of their respective metabolism on the efficacy of silver against each microorganism.

Furthermore, other factors may influence the antimicrobial potential of AgNP [34]. For instance, how the compound containing silver interacts with the microorganisms would dependent on the characteristics of the AgNP formed, as well as the chemical and physical changes that may occur when they are added to the medium of interest [33]. In general, for the synthesis of AgNP, AgNO_3_ is used as source of silver, water or ethanol as solvent, and sodium borohydride or sodium citrate as reducing agent [39]. Fabricated under similar conditions, the AgNP would have a negative surface charge [33,40], and this fact is noteworthy to elucidate the lower effectiveness of the compounds against *S. mutans*. Bacteria have a negative outer membrane charge [41] and the electrostatic attraction may have been hampered, and hence the action of the AgNP associated or not with CaGP on the *S. mutans* was diminished. On the other hand, apart from fungi present a neutral surface charge [41] and might enhance the attraction of AgNP, the presence of phospholipid components, which contain phosphate groups, may have improved the antimicrobial activity of silver by targeting these sites [42,43]. Indeed, the control of AgNP reduced by sodium citrate showed a lower amount of ions (107.2 µg Ag^+^/mL) than the control that was produced using sodium borohydride (576.2 µg Ag^+^/mL), and it was more effective against *C. albicans*, suggesting an antifungal potential of AgNP by itself, which may have afforded the disruption of the *C. albicans* cell membrane by damaging the inner layers of the cell wall, increasing their permeabilization and then allowing for the passage of these particles to into the cell.

On the contrary, against *S. mutans* plaktonic cells, Ag^+^ may have played a preponderant role, particularly in view of the MIC values that are found for AgNO_3_ (21.2 µg/mL) when compared to those for AgNP, regardless of the reducing agent that is used in the reaction (250 and 125 µg/mL, respectively, for AgNP (Na_3_C_6_H_5_O_7_) and AgNP (NaBH_4_)). Noteworthy was the effect that was produced against *S. mutans* when CaGP was associated with AgNP (Table 1). CaGP afforded an increment in the silver activity and it could be related to the acidogenic and acidic characteristic of *S. mutans*, acting CaGP probably as a buffer, and hence might have prevented the proliferation of the cells in the medium [44,45,46]. So that, the CaGP buffer activity and the highest amount of Ag^+^ ions could account for the better effectiveness of the samples of C group against that gram positive bacteria tested.

## 4. Materials and Methods

### 4.1. Synthesis of Silver-Calcium Glycerophosphate (Ag/CaGP) Nanocomposites

Ag/CaGP nanocomposites were synthesized at the Interdisciplinary Laboratory of Electrochemistry and Ceramics of the Chemistry Department in Federal University of São Carlos. Initially, the commercial form of calcium glycerophosphate (80% β-isomer and 20% rac-α-isomer, CAS 58409-70-4, Sigma-Aldrich Chemical Co., St. Louis, MO, USA) was acquired and was nanoparticulated using a ball mill for 24 h at 120 rpm, obtaining nanoparticles of approximately 10 nm. Then, two chemistry methods were employed for the synthesis. The first method was employed using sodium borohydride as reducing agent (NaBH_4_, Sigma-Aldrich Chemical Co., St. Louis, MO, USA) and was based on the methodology that was proposed by Miranda et al. [29]. The synthesis was carried out in an alcoholic medium (isopropanol) or deionized water. For this, suspensions containing 5 g of CaGP and silver nitrate (AgNO_3_ Merck KGaA, Darmstadt, Hessen, Germany) at 0.85 or 0.085 g were prepared in the presence of 0.5 mL of a surfactant (ammonium salt of polymethacrylic acid (NH-PM), Polysciences Inc., Warrington, PA, USA) (Table 1). Then, NaBH_4_ (0.015 g) was added to each suspension, which caused the reduction of Ag^+^ to metallic silver nanoparticles in the presence of CaGP. The molar stoichiometric ratio between Ag^+^ and NaBH_4_ was 1:1.26, respectively. The second method was based on that proposed by Turkevich et al. [47] and Gorup et al. [48] (2011, p. 355). The reducing agent of AgNO_3_ was sodium citrate (Na_3_C_6_H_5_O_7_, Merck KGaA) and the stoichiometric ratio of each compound was, respectively, 1:3. Thus, in a flask containing 100 mL of deionized H_2_O 5 g of CaGP was added following of 0.5 mL NH-PM and 1.4 g of Na_3_C_6_H_5_O_7_. This mixture was kept under magnetic stirring and heating. After reaching 95 °C temperature, AgNO_3_ was added and this suspension was maintained stirring for 30 min until occurring the color change, which qualitatively indicated the formation of AgNP. Controls containing only the reducing agents and surfactant, and AgNP produced by both reducing were also prepared.

### 4.2. Characterization of Ag-CaGP Nanocomposites

In order to demonstrate the presence of AgNP and CaGP in the compounds, the UV-Vis absorption spectroscopy was employed. The measure is based on the phenomenon of plasmon resonance band, as observed in metallic nanoparticles. Thus, UV-Vis spectra of Ag-CaGP nanocomposites were obtained from aqueous solutions poured out in a commercial quartz cuvette with 1 cm optical path using a spectrophotometer (Shimadzu MultSpec-1501 spectrophotometer; Shimadzu Corporation, Tokyo, Japan) at 300 to 800 nm. Water was used as blank.

After a drying step, the resulting powder, Ag-CaGP, was subjected to X-ray diffraction (XRD) phase characterization using Cu Kα radiation (λ = 1.5406 Å), generated at a voltage of 30 kV and a current of 30 mA with continuous sweep in the range of 5° < 2θ < 80°, at a scan rate of 2°/min (Diffractometer Rigaku DMax-2000PC, Rigaku Corporation, Tokyo, Japan). The particles morphology was also characterized by scanning electron microscopy (SEM) on a Zeiss Supra 35VP microscope (S-360 Microscope, Leo, Cambridge, MA, USA), with field emission gun electron effect (FEG-SEM) operating at 10 kV. A drop of each sample were added with a micropipette and deposited on silicon metal plate (111) and dried at 40 °C for 2 h. With this technique, we can identify in the synthesized biomaterials the presence of silver, oxygen, silicon, phosphate, and calcium, which were artificially colored (Figure 3 and Figure 4).

### 4.3. Minimum Inhibitory Concentration (MIC)

The MIC values for each sample were determined through the microdilution method and followed the Clinical Laboratory Standards Institute guidelines (CLSI, documents M27-A2 and M07-A9). *Candida albicans* (ATCC 10231) was cultivated on Sabouraud Dextrose Agar (SDA, Difco, Le Pont de Claix, France) and *S. mutans* (ATCC 25175) on Brain Heart Infusion Agar (BHI, Difco, Le Pont de Claix, France). Inocula from 24 h cultures on the respective media were adjusted to a turbidity equivalent to a 0.5 McFarland standard in saline solution (0.85% NaCl). This suspension was diluted (1:5) in saline solution, and afterwards diluted (1:20) in RPMI 1640 or BHI. Initially, the Ag-CaGP nanocomposite was diluted in deionized water in a geometric progression, from 2 to 1024 times. Afterwards, each Ag-CaGP nanocomposite concentration obtained previously was diluted (1:5) in RPMI 1640 medium (Sigma-Aldrich) for *C. albicans* and in BHI for *S. mutans*. The final concentrations of Ag-CaGP nanocomposite in the dispersion ranged from 5 to 0.01 mg/mL. Each inoculum (100 µL) was added to the respective well of microtiter plates containing 100 µL of each specific concentration of the Ag-CaGP nanocomposite solution. The microtiter plates were incubated at 37 °C, and the MIC values were determined visually as the lowest concentration of Ag-CaGP with no microorganism growth after 48 h for *C. albicans* and 24 h for *S. mutans*. All of the assays were repeated in triplicate on three different occasions.

### 4.4. Determination of Ag^+^ Concentration

The evaluation of Ag^+^ concentration in Ag-CaGP and AgNP, as obtained by both reducing agents, was determined by a specific electrode 9616 BNWP (Thermo Scientific, Beverly, MA, USA) coupled to an ion analyzer (Orion 720 A^+^, Thermo Scientific, Beverly, MA, USA). A 1000 µg/mL silver standard was prepared placing 1.57 g of dried AgNO_3_ into a 1 L volumetric flask containing deionized water. This solution was stored in an opaque bottle in a dark location and diluted in deionized water at the moment of dosage in order to achieve the standard concentrations used. Thus, the combined electrode was calibrated with standards containing 6.25 to 100 µg Ag/mL at the same conditions of samples. A silver ionic strength adjuster solution (ISA, Cat. No. 940011) that provides a constant background ionic strength was used (1 mL of each sample/standard: 0.02 mL ISA).

## 5. Conclusions

In conclusion, the synthesis that is proposed in this study promoted the anchorage of AgNP with the CaGP, and the nanocomposites produced using sodium citrate as reducing agent were effective against both of the microorganisms tested. Also, the highlight of our study was that the addition of CaGP to AgNP expressively reduced the MIC values when it was compared with the MIC values of AgNP by itself. These promising results strongly encourage further studies with the purpose of producing biomaterials with antimicrobial and remineralizing functions in the near future, particularly in the dental field.

## Figures and Tables

**Table 1:** Values of minimum inhibitory concentration (MIC) of the nanocompounds based on µg of AgCaGP mL^−1^ and on µg of Ag mL^−1^ in each ones, synthesized using sodium borohydride (Group B) and sodium citrate (Group C), and silver ions concentration (µg Ag^+^/mL) in all nanocompounds tested.

GROUP	Ag %/CaGP Form	Solvent	MIC (µg AgCaGP mL^−1^/µg Ag mL^−1^)		µg Ag^+^/mL
			*C. albicans*	*S. mutans*	
B1	1/M	I	>1600/>16	>1600/>16	2.83
B2	1/N	I	400–1600/0.40–16	>1600/>16	4.46
B3	10/M	I	400–800/40–80	>1600/>16	10.81
B4	10/N	I	100–200/10–20	>1600/>16	63.34
B5	1/M	W	>1600/>16	>1600/>16	0.44
B6	1/N	W	>1600/>16	>1600/>16	2.76
B7	10/M	W	>1600/>16	>1600/>16	5.97
B8	10/N	W	>1600/>16	>1600/>16	15.63
C1	1/M	W	156.2–312.5/1.56–3.12	1250/12.5	305.43
C2	1/N	W	156.2–312.5/1.56–3.12	1250/12.5	168.14
C3	10/M	W	39.0/3.9	312.5–625/31.2–62.5	506.73
C4	10/N	W	19.5–39.0/1.9–3.9	156.2–312.5/15.6–31.2	487.95

**Table 2:** Values of minimum inhibitory concentrations (MIC) (µg/mL) and silver ions (Ag^+^) (µg Ag^+^/mL) of the control solutions: AgNP reduced by sodium citrate (Na_3_C_6_H_5_O_7_), and by sodium borohydride (NaBH_4_); silver nitrate (AgNO_3_); nanoparticulated CaGP (CaGP-nano), and CaGP in commercial form (CaGP-commercial); sodium citrate and surfactant (Na_3_C_6_H_5_O_7_+NH-PM), and sodium borohydride and surfactant (NaBH_4_+NH-PM).

Controls	MIC		Ag^+^
	*C. albicans*	*S. mutans*	
AgNP (Na_3_C_6_H_5_O_7_)	7.8	250	107.2
AgNP (NaBH_4_)	62.5	125	576.2
AgNO_3_	5.3	21.2	-
CaGP-nano	>5000	>5000	-
CaGP-commercial	>5000	>5000	-
Na_3_C_6_H_5_O_7_+NH-PM	>400	>400	-
NaBH_4_+NH-PM	>1500	>1500	-

## Supplementary Materials

The following are available online at http://www.mdpi.com/2079-6382/7/3/52/s1, Figure S1: UV–Visible spectrum of Ag-CaGP nanocomposites, Figure S2: X-ray diffraction pattern of Ag-CaGP nanocomposites, Figure S3: TEM images of B4 Ag-CaGP nanocomposite, Figure S4: SEM images and energy-dispersive X-ray sprectroscopy (EDS) mapping in 2D elements issuance Si Kα, O Kα, P Kα,Ca Kα and Ag Kα false color. Analysis of the distribution of silver nanoparticles in the Ag-CaGP nanocomposites B1, B2 and B3, Figure S5: SEM images and EDS mapping in 2D elements issuance Si Kα, O Kα, P Kα,Ca Kα and AgKα false color. Analysis of the distribution of silver in the Ag-CaGP nanocomposites B5, B6 and B7, Figure S6: SEM images and EDS mapping in 2D elements issuance Si Kα, O Kα, P Kα,Ca Kα and Ag Kα false color. Analysis of the distribution of silver nanoparticles in the Ag-CaGP nanocomposites C1, C2 and C3.
