# Supplementary material for: Nanosynthesis of Silver-Calcium Glycerophosphate: Promising Association against Oral Pathogens
Source: Antibiotics (Basel). 2018 Jun 27;7(3):52. doi: 10.3390/antibiotics7030052 (PMC6163287; doi:10.3390/antibiotics7030052)
Supplement: Supplementary file 1 [file antibiotics-07-00052-s001.pdf]

Electronic Supplementary Information (ESI)

Nanosynthesis of silver-calcium glycerophosphate: promising association against oral pathogens

Gabriela Lopes Fernandes[1], Alberto Carlos Botazzo Delbem[2], Jackeline Gallo do Amaral[2], Luiz Fernando Gorup[3,4], Renan Aparecido Fernandes[1,5], Francisco Nunes de Souza Neto[3], José Antonio Santos Souza[2], Douglas Roberto Monteiro[6] Alessandra Marçal Agostinho Hunt[7], Emerson Rodrigues Camargo[3], Debora Barros Barbosa[1*]

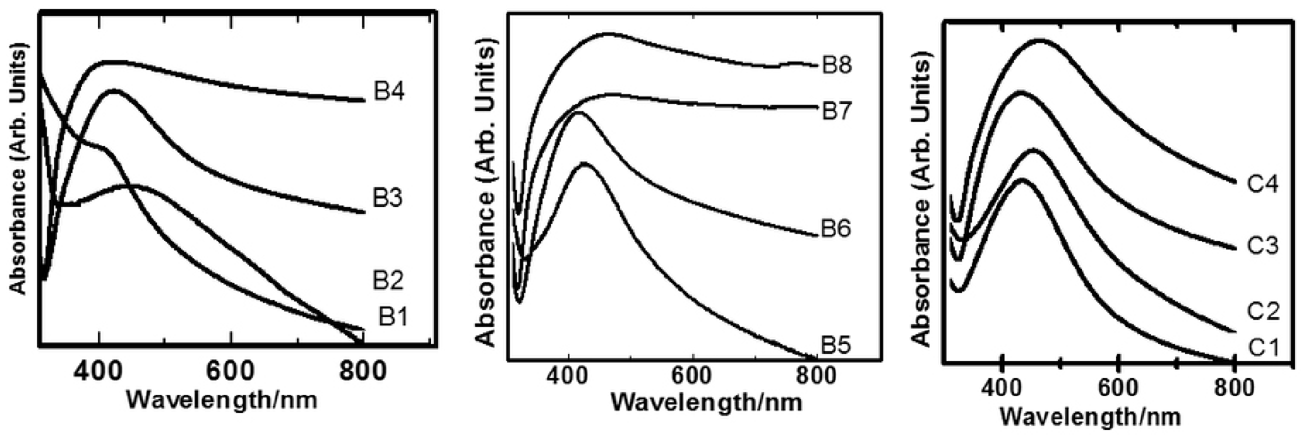

Figure S1: UV–Visible spectrum of Ag-CaGP nanocomposites.

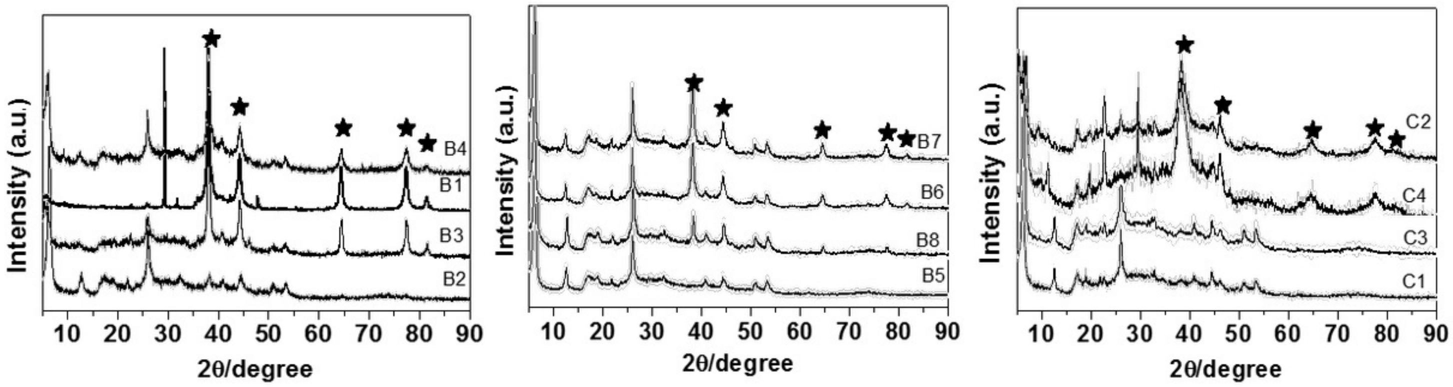

Figure S2: XRD pattern of Ag-CaGP nanocomposites.

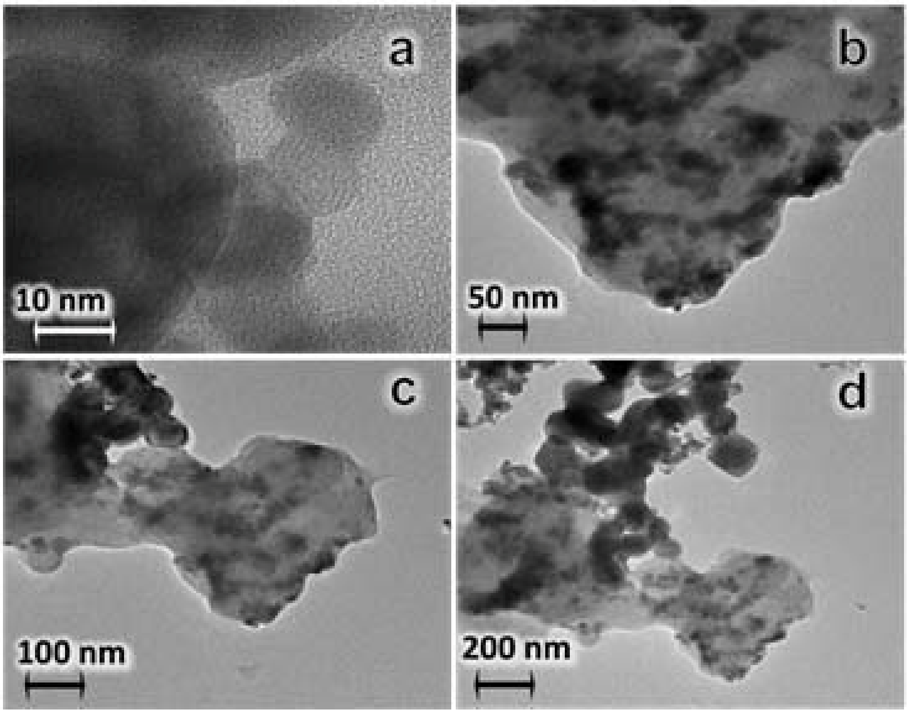

Figure S3: TEM images of B4 Ag-CaGP nanocomposite.

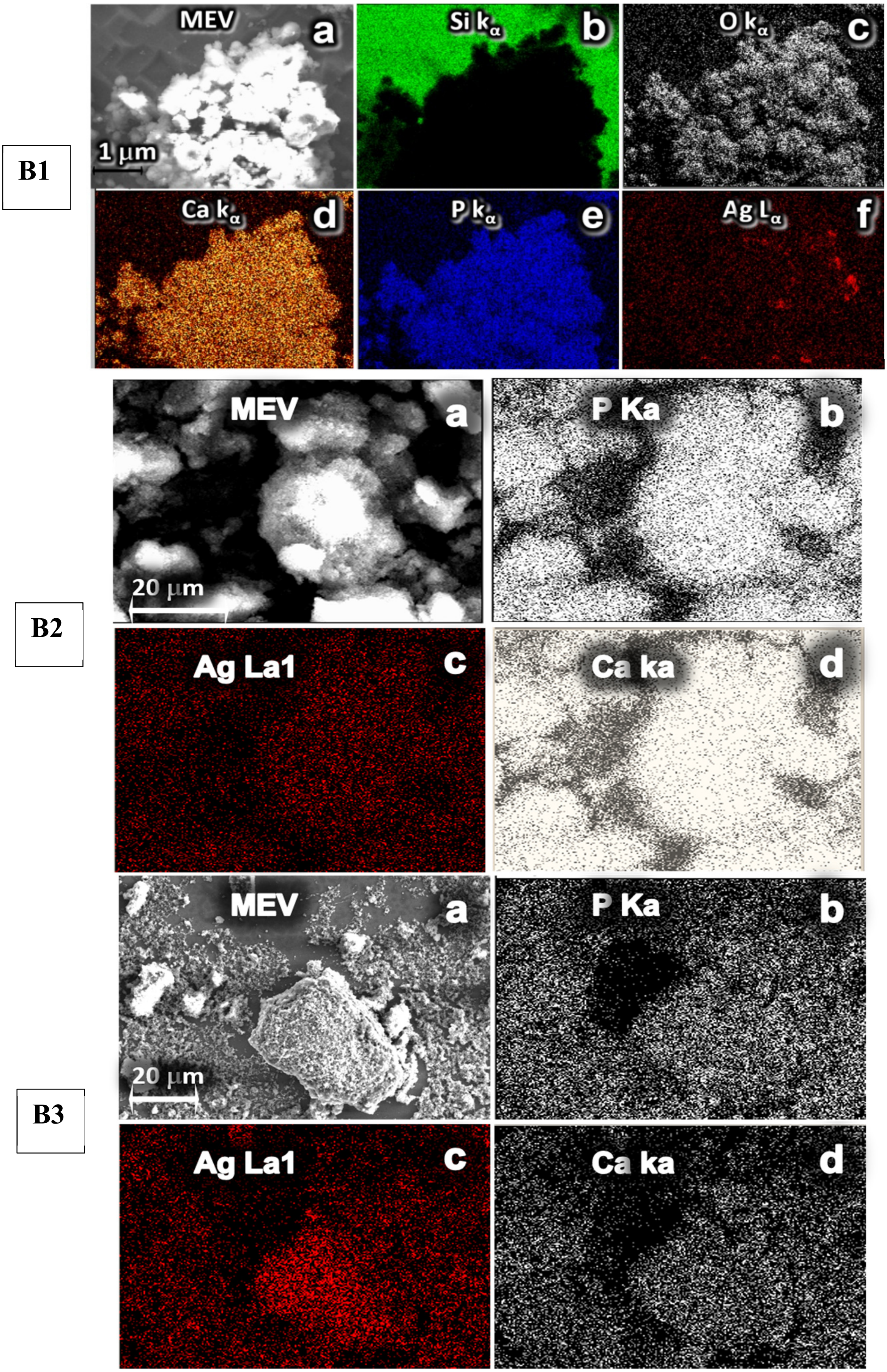

Figure S4: SEM images and energy-dispersive X-ray sprectroscopy (EDS) mapping in 2D elements issuance Si Kα, O Kα, P Kα,Ca Kα and Ag Kα false color. Analysis of the distribution of silver nanoparticles in the Ag-CaGP nanocomposites B1, B2 and B3

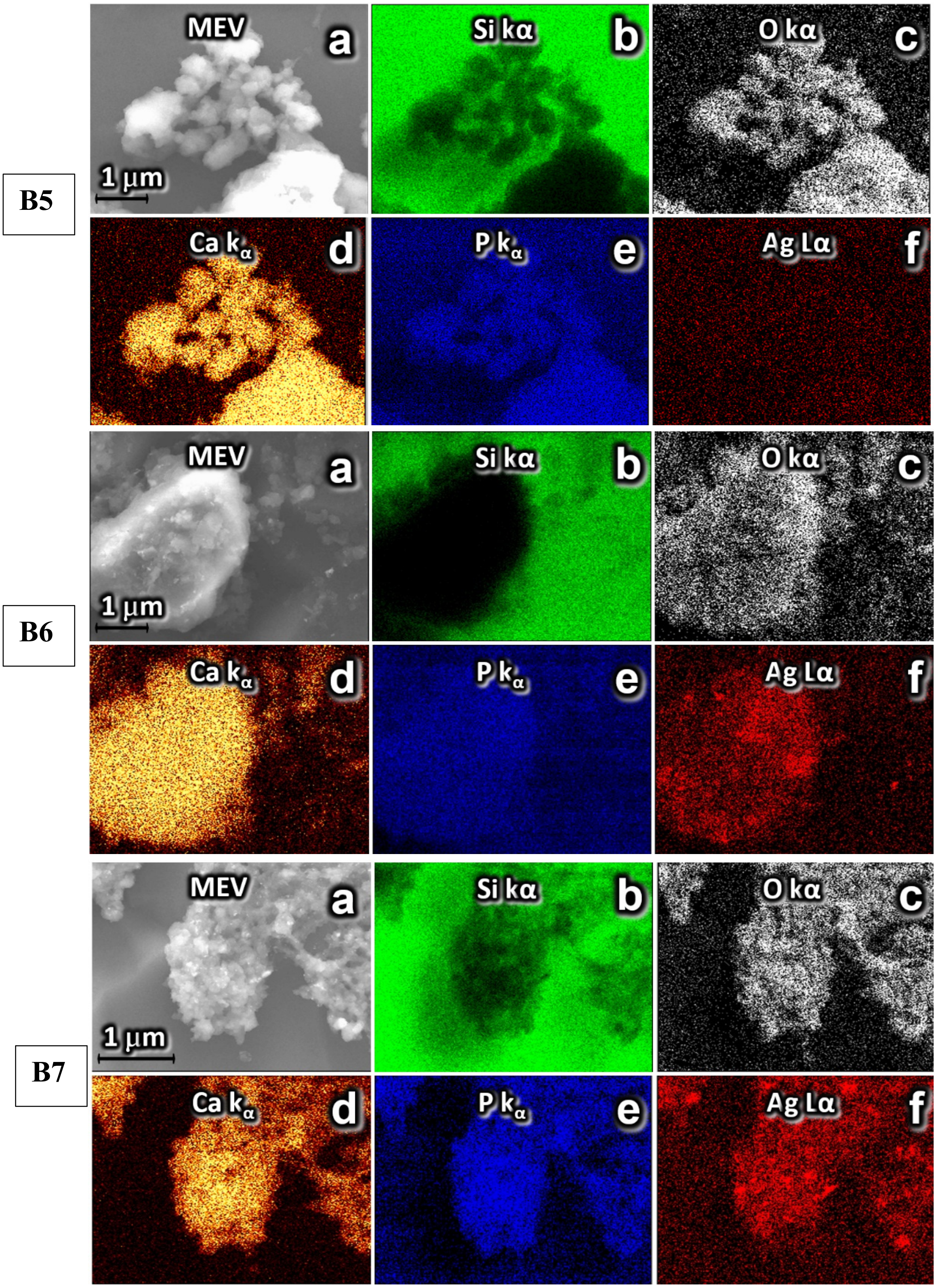

Figure S5: SEM images and EDS mapping in 2D elements issuance Si Kα, O Kα, P Kα,Ca Kα and AgKα false color. Analysis of the distribution of silver in the Ag-CaGP nanocomposites B5, B6 and B7

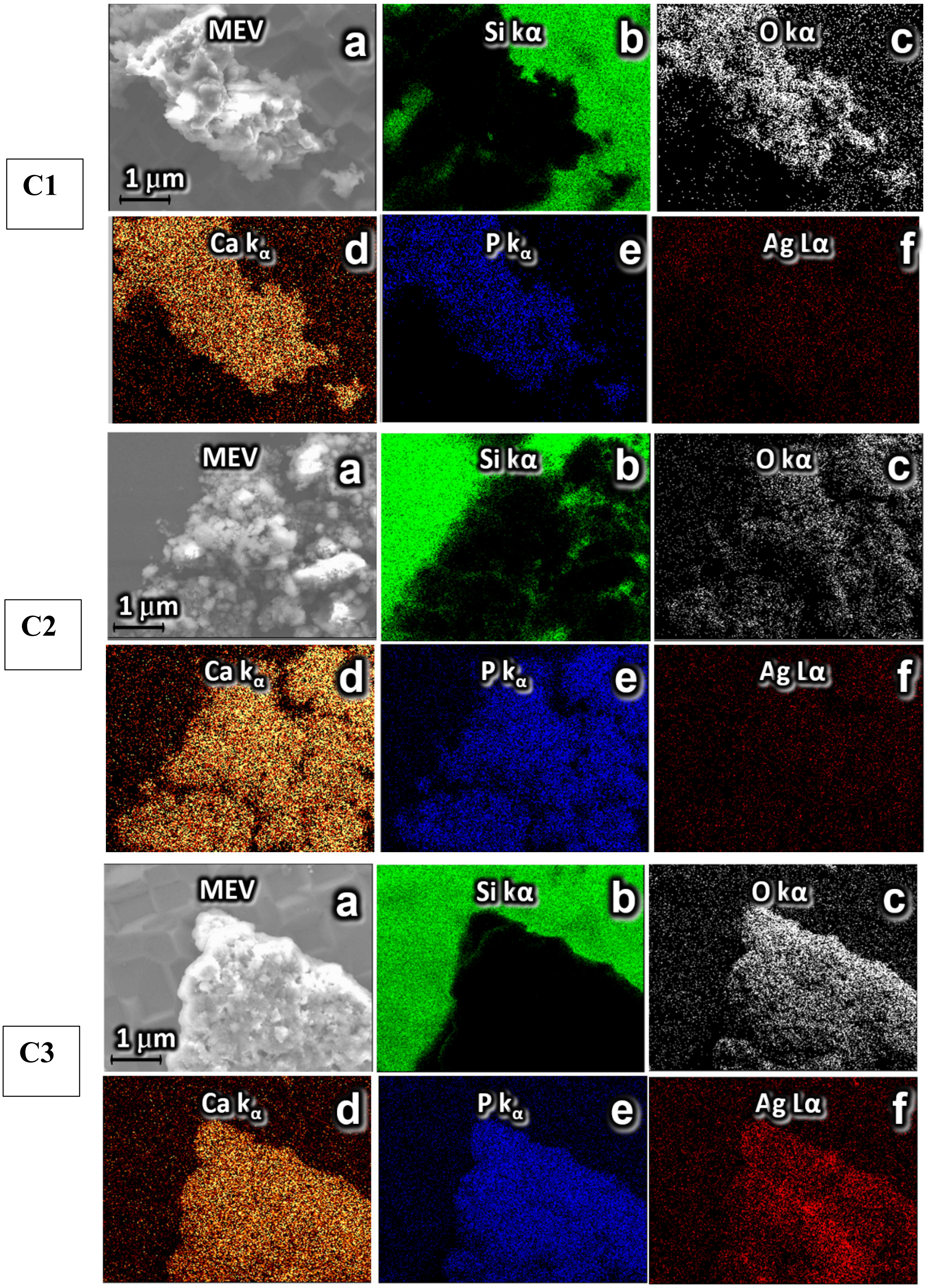

Figure S6: SEM images and EDS mapping in 2D elements issuance Si Kα, O Kα, P Kα,Ca Kα and Ag Kα false color. Analysis of the distribution of silver nanoparticles in the Ag-CaGP nanocomposites C1, C2 and C3.
